# Advancing an account of hierarchical dual-task control: A focused review on abstract higher-level task representations in dual-task situations

**DOI:** 10.1177/17470218241295524

**Published:** 2024-11-18

**Authors:** Patricia Hirsch, Iring Koch

**Affiliations:** Cognitive and Experimental Psychology, Institute of Psychology, RWTH Aachen University, Aachen, Germany

**Keywords:** Dual tasks, PRP effect, task-pair switch costs, dual-task representation

## Abstract

Dual tasks are a common phenomenon in everyday life. In dual-task contexts, we perform two-component tasks in temporal overlap, which usually results in impaired performance in one or both of these component tasks relative to single-task contexts. Numerous studies have examined dual-task interference at the level of response selection, but only a few studies have addressed the cognitive representation of a dual task and the cognitive mechanisms controlling these representations. The present review outlines recent empirical findings and theoretical developments concerning these two issues. In detail, the review focuses on different components of a cognitive dual-task representation, including the representation of component-task-specific information (i.e., information about the goal and stimulus-response mapping of a component task), the representation of component-task order information (i.e., information about the order in which the component tasks have to executed), and the representation of dual-task identity information (i.e., information about which two-component tasks have to be performed). A particular emphasis is placed on the cognitive representation of dual-task identity information, which is examined in a recent research line employing the task-pair switching logic as an empirical approach. By conceptualising a dual-task representation as a hierarchical multi-component representation, the review integrates the research line on the cognitive representation of dual-task identity information with the research lines on the representation of component-task-specific information and component-task order information. Based on this conceptualisation, the review provides a new theoretical contribution to dual-task research and highlights an integrative perspective on the different components of cognitive dual-task representations.

## Introduction

In everyday life, we often face dual-task demands. For instance, while listening to a talk, we take notes or answer urgent messages on the cell phone. However, research showed that when performing two tasks in temporal overlap, performance typically decreases in one or both tasks compared to when performing the tasks in a single-task situation (see, e.g., Fischer & Janczyk, 2022; Janczyk & Kunde, 2020; Koch et al., 2018; Pashler, 1994, for reviews). The performance decline is usually reflected in both reaction times (i.e., RTs) and error rates, indicating that in dual-task situations, more time is needed to complete the tasks, and the vulnerability to errors is greater than in single-task situations. This performance decline has often been attributed to capacity limitations concerning central processes (see, e.g., Fischer & Plessow, 2015; Janczyk & Kunde, 2020, for reviews). Central processes intervene between sensory and motor processes and represent processes of decision and response selection (e.g., Pashler, 1994).

Numerous studies have been conducted to characterise this central capacity limitation, but comparatively fewer studies have explored the nature of a cognitive dual-task representation and the interference that arises during the selection of these representations (see, e.g., Fischer & Janczyk, 2022; Koch et al., 2018; Pashler, 1994, for reviews). Cognitive task representations are a key concept in theoretical accounts of cognitive psychology, and the perspective on cognitive task representations is foundational for research in other multi-tasking domains, such as task switching, where the principles of human information processing are explored by analysing the performance cost incurred when switching between single tasks (e.g., Kleinsorge & Heuer, 1999; Philipp & Koch, 2010; Vandierendonck et al., 2008; see also Koch et al., 2018, for a review). This perspective is, however, still relatively new to dual-task research.

### The aim and structure of the review

The present review provides an overview of dual-task studies that focus on the transfer of abstract task representations from one dual-task trial to another, with the aim of gaining insights into the nature of cognitive dual-task representations and the cognitive processes that govern their selection. Prior to reviewing this literature, we first present a brief overview of the basic empirical methods, behavioural performance measures, and theoretical models that are often used to examine processing limitations in dual-task contexts. This will illustrate that the nature of cognitive dual-task representations and the cognitive mechanisms underlying their selection have not been taken into account in traditional dual-task studies. Second, we outline recent empirical findings and theoretical developments concerning the components of a dual-task representation and the cognitive processes supporting their selection. In this section, we address the cognitive representation of component-task-specific information (i.e., information about the goal and S-R mapping of a component task), followed by the cognitive representation of component-task order information (e.g., information about the order in which the component tasks have to be executed). After that, we provide a detailed overview of research on the cognitive representation of dual-task identity information (i.e., information needed to identify the relevant component tasks of a dual task), which is the primary focus of the present review. In the third section, we generate an overarching theoretical contribution by proposing to conceptualise the cognitive representation of a dual task as a hierarchical dual-task representation which incorporates various components. Finally, we elaborate on the question of what we can learn from the reviewed research regarding transfer, followed by a summary of the major conclusions of this review.

## Dual-task interference at the level of response selection

### Empirical approaches to study dual-task interference

Dual-task studies differ mainly with regard to the experimental design, falling into two classes of empirical paradigms (see e.g., Fischer & Janczyk, 2022; Koch et al., 2018, for reviews). Some studies contrasted dual-task performance with single-task performance, whereas other studies compared dual-task performance across conditions with different degrees of temporal overlap between two tasks.

#### Dual-task versus single-task approach

In the dual-task versus single-task approach, subjects perform two tasks simultaneously, or they perform the tasks separately in a single-task context. The basic finding is that for both tasks, performance, as evident in RTs and error rates, is worse in dual-task conditions than in single-task conditions, reflecting dual-task costs (e.g., Halvorson et al., 2013; Hensen et al., 2024).

For instance, Huestegge and Koch (2013, Experiment 3) presented a tone either to the left or right ear and a square to the left and right of a fixation cross that appeared in the middle of the screen. The tone was linked to both a saccade task and a manual task. In the saccade task, participants had to perform a saccade to the spatially compatible square, and in the manual task, they had to press a spatially compatible key. In the dual-task condition, participants performed both tasks simultaneously, whereas in the single-task condition, they performed only one task. The authors found that performance was worse in both tasks when the tasks had to be executed simultaneously than when they were performed in a single-task context (dual-task costs of 42 ms). Even larger dual-task costs were observed when participants were instructed to cross their hands and to press the key with the hand that corresponds to the tone-presentation side (197 ms; Huestegge & Koch, 2009, Experiment 3).

#### Psychological refractory period approach

**Figure 1.**
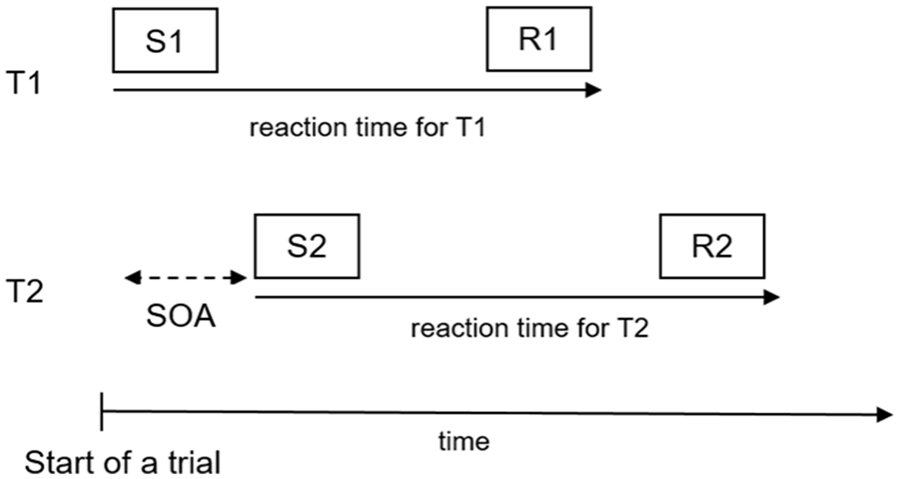
The psychological refractory period (PRP) paradigm. *Note.* T1 = Task 1, T2 = Task 2, S1 = stimulus for T1, S2 = stimulus for T2, R1 = response for T1, R2 = response for 2, SOA = stimulus onset asynchrony.

In contrast to the dual-task versus single-task approach, in the psychological refractory period (PRP) paradigm, participants perform two tasks in each trial, and the extent to which the processing of the two tasks overlaps in time is systematically manipulated (e.g., Telford, 1931; Welford, 1952; see e.g., Koch et al., 2018; Pashler, 1994 for reviews). The tasks are referred to as component tasks and are usually simple two-choice RT tasks, which are linked to separate stimuli and responses. The degree of temporal task overlap is manipulated based on the stimulus-onset-asynchrony (SOA), which is the time interval between the onset of the stimulus for T1 (S1) and the presentation of the stimulus for T2 (S2; see Figure 1). Hence, short SOAs result in a considerable temporal overlap in T1 and T2 processing, whereas long SOAs lead to less or even no temporal overlap.

The SOA manipulation usually does not affect RT for T1 (RT1), whereas RT for T2 (RT2) increases as SOA decreases, an effect referred to as the PRP effect (e.g., Hirsch et al., 2019; Koch & Jolicoeur, 2007; Rieger et al., 2021; see Fischer & Janczyk, 2022; Pashler, 1994, for reviews; see also Strobach, Schütz, & Schubert, 2015, for a review about T1 performance). This effect indicates that T2 performance gets worse with increasing temporal overlap in task processing.

For instance, in a study by Jentzsch and colleagues (2007), participants were presented with a tone, and after a random SOA of either 100 ms, 400 ms, or 800 ms, a letter appeared on the screen. T1 was to categorise the tone as low or high in pitch and T2 to categorise a letter as a X or O. The authors observed that RT2 decreased with increasing SOA, reflecting a PRP effect. In detail, the mean RT2 was 737 ms for the SOA of 100 ms, 525 ms for the SOA of 400 ms, and 444 ms with the SOA of 800 ms. Note that even though the PRP effect has been found to be more consistent in RTs, there are also some studies reporting the PRP effect for the error rates (e.g., Hosang et al., 2018; Mittelstädt et al., 2022).

#### Summary

Two main experimental approaches have been used to study dual-task interference. In the single versus dual-task approach, task performance is contrasted across single-task and dual-task conditions, whereas in the PRP paradigm, there are two tasks in each trial, and performance is compared between short and long SOAs. Both approaches result in dual-task interference, as reflected in dual-task costs and the PRP effect. These two behavioural measures are affected by several factors, such as ideomotor compatibility, practice, and preparation (e.g., Greenwald, 1972; Liepelt et al., 2011; Lyphout-Spitz et al., 2024; Maquestiaux et al., 2020; see e.g., Koch et al., 2018; Strobach, 2020 for reviews).

### Theoretical accounts on dual-task interference phenomena

Models on dual-task interference phenomena share the notion that the processing of each component task is organised into three serial processing stages, including perceptual processing (i.e., stimulus encoding), response selection (i.e., translation of the stimulus to its response), and response execution. The models can be categorised into structural response-selection bottleneck models (e.g., Pashler, 1994), capacity-sharing models (e.g., Tombu & Jolicoeur, 2003), crosstalk models (e.g., Hommel, 1998), and strategic response-selection bottleneck models (e.g., Logan & Gordon, 2001; Meyer & Kieras, 1997a, 1997b).

#### Structural response-selection bottleneck models

According to structural response-selection bottleneck models (see, e.g., Pashler, 1994, for a review), the processing stages of perceptual processing, response selection, and response execution occur for each component task separately, so that performing two tasks reflects two independent processing streams. Perceptual and response-execution stages can proceed in parallel for two tasks, whereas processing at the central stage of response selection takes place serially for two tasks, representing a structural all-or-none bottleneck (e.g., Koch et al., 2018).^1^ This notion implies that response selection is a capacity-limited process and that the capacity can only be allocated to one task at a time. Consequently, response selection for T2 has to wait until the completion of the response-selection process for T1, resulting in dual-task interference.

#### Capacity-sharing models

Similar to structural response-selection bottleneck models, capacity-sharing models posit that the response selection stage is the locus of structural capacity limitations. However, contrary to structural response-selection bottleneck models, the limited capacity can be allocated in a graded manner to the component tasks, thereby enabling response selection to occur simultaneously for both tasks (e.g., Tombu & Jolicoeur, 2003; see also Mittelstädt et al., 2022; Schaaf et al., 2023). Dual-task interference arises because the amount of capacity allocated to a task determines the processing rate of a task, and in dual-task contexts with considerable temporal task overlap, the limited capacity has to be shared for a longer time period than in dual-task contexts with less temporal task overlap.

#### Crosstalk models

Compared to the models described so far, crosstalk models propose a theoretically distinct source of dual-task interference. These models theorise that the processing streams of the two component tasks are not fully independent, and that dual-task interference is related to overlap in the task content across tasks (e.g., Lien & Proctor, 2002). More precisely, dual tasks lead to crosstalk when physical features or conceptual dimensions overlap across the stimuli and/or responses of the two component tasks, meaning that there is an overlap in their processing codes (e.g., Navon & Miller, 1987). This is, for instance, the case when T1 requires manual responses with a left or right key and T2 the vocal responses “left” or “right” (e.g., Hommel, 1998). Crosstalk refers to any unintentional transmission of information, such as stimulus or response codes, from the processing stream of one component task to that of the other component task (e.g., Huestegge & Koch, 2009; see also Koch et al., 2018). The information transmission can impair performance in a component task by priming an inadequate stimulus interpretation and leading to confusion between the response codes of the two component tasks. For example, when T2 requires the vocal response “left,” and the correct response for T1 is a right key press, then the response features do not overlap between T1 and T2. This can lead to confusion between the response codes of the two component tasks and impair T1 performance, when there is temporal overlap in T1 and T2 processing (e.g., Hommel, 1998; Janczyk et al., 2017; Koch, 2009; Koch & Prinz, 2002; Miller, 2006).

#### Strategic response-selection bottleneck models

Strategic response-selection bottleneck models hold that dual-task interference is attributable to a strategic decision for serial response selection (e.g., executive-process/ interactive-control, EPIC, architecture by Meyer & Kieras, 1997a, 1997b executive control of visual attention model by Logan & Gordon, 2001). These models postulate that parallel response selection is generally possible, but that serial processing is more efficient and, therefore, the predominant multi-tasking strategy (see, e.g., Fischer & Plessow, 2015, for a discussion). For instance, in the framework of the EPIC architecture, the two-component tasks are represented by separate “production rule” sets, which contain a task goal and the if-then rules needed to achieve the task goal. An executive process rule set coordinates the processing of the two component tasks in accordance with the current dual-task demands. For this purpose, the executive process rule set determines “lock-out” points in T2, where T2 processing is suspended, and “lock-in” events in T1 that allow T2 processing to continue. A key notion is that these “lock-out” and “lock-in” points are flexibly implemented at the stages of perceptual processing, response selection, and response execution. To mitigate processing conflicts, such as the above-mentioned crosstalk effects, the “lock-out” point in T2 can be set prior to the response selection in T2 and the “lock-in” point in T1 after the completion of the response-selection process in T1. In addition to this strategy, the executive process rule set can follow an interleaved scheduling strategy, in which tasks are performed in parallel with brief suspensions of a task when parallel processing would cause crosstalk between tasks.

#### Summary

Even though the exact nature of dual-task interference is not yet fully understood, there is a consensus that dual-task interference arises at the level of response selection. Accordingly, numerous studies have been conducted to examine the effects of dual-task demands on response selection (e.g., Janczyk et al., 2014; Mattes et al., 2021). Substantially less research effort has been put into studying the nature of a cognitive dual-task representation and the interference arising during the selection of these representations.

## In search of the cognitive representation of dual tasks and the cognitive processes supporting their selection

The notion that dual-task interference arises at the level of response selection has been a fruitful basis for a substantial body of empirical and theoretical studies. However, in this venerable research tradition, two issues have been mostly neglected that refer to the transfer of abstract task representations in dual-task performance.

The first issue relates to the question of how a dual task is cognitively represented. The cognitive representation of a task is a mental representation of the cognitive and motor demands of a task which has to be activated in working memory to perform a task (e.g., Kiesel et al., 2010; Vandierendonck et al., 2010 for a discussion). It contains all information (e.g., stimuli, responses, S-R mappings, and task goals), enabling task processing from stimulus encoding to responding (e.g., Vandierendonck et al., 2008). In a more formal sense, the cognitive representation of a task entails a set of programmable parameters that bias the processing of sensory input and responding in accordance with the current task goals (e.g., Logan & Gordon, 2001; Schneider & Logan, 2014).^2^

The second issue refers to the cognitive processes supporting the selection of dual-task representations. The correct execution of a task presupposes that its cognitive representation is the most active among all other cognitive task representations (e.g., Grange & Houghton, 2014). As at the theoretical level, performance costs in dual-task contexts have been primarily attributed to interference at the level of response selection, the majority of studies have neglected interference arising during the selection of more abstract dual-task representations.

Both of these issues can be explored by analysing sequential effects across dual-task trials. Such analyses answer the question of whether there are automatic carry-over effects of the dual-task demands in the previous trial on the performance in a current dual-task trial. Carry-over effects reflect a type of automatic transfer of abstract cognitive task representations. Transfer refers to the application of knowledge, skills, and information learned in a previous task to a novel task (e.g., Barnett & Ceci, 2002). It can result in transfer benefits when previous experience with a task facilitates the completion of a current task, but it can also result in transfer costs when previous experience hampers the completion of a current task. Applied to abstract dual-task representations, transfer benefits occur when the cognitive dual-task representation needed in the previous dual-task trial is still activated and can be used again in the subsequent dual-task trial. This is the case when the processing demands are constant across two consecutive dual-task trials. In contrast, when the previous dual-task representation cannot be applied to the next dual-task trial, the transfer of a cognitive dual-task representation can even result in transfer costs, as evident in performance costs. This is the case when the processing demands differ across two consecutive dual-task trials.

Existing dual-task studies examined carry-over effects of different components of dual-task representations, such as component-task-specific information (e.g., information about the goal and S-R mapping of a component task), component-task order information (e.g., information about the order in which the component tasks have to be executed), and dual-task identity information (i.e., information needed to identify the relevant component tasks of a dual task). The studies showed that dual-task performance is worse when the cognitive representation of component-task-specific information, component-task order information, and/or task-identity information used in a previous dual-task trial cannot be applied again in the subsequent dual-task trial than when this information remains constant across two subsequent dual-task trials than when this information remains constant across two subsequent dual-task trials (e.g., Hirsch et al., 2017; Lien et al., 2003; Luria & Meiran, 2003). These performance costs reflect a type of switch costs and are interpreted as a marker of interference at the level of selecting and implementing cognitive dual-task representations. In contrast, dual-task costs and the PRP effect are usually thought to reflect interference at the level of response selection within a PRP trial, and, thus, refer to a different processing level (see e.g., Fischer & Janczyk, 2022; Fischer & Plessow, 2015; Koch et al., 2018; Pashler, 1994, for reviews). In the following, we outline the empirical findings and theoretical developments concerning these three components of a cognitive dual-task representation.

### Representations of component-task-specific information and their control

Several studies permit conclusions regarding the nature of a dual-task representation, despite the fact that they were originally conducted to examine other aspects of dual-task performance. For example, this is the case for PRP studies which manipulated the task switching requirements at the level of T1 and T2 within a trial (e.g., Band & van Nes, 2006). These studies provide insights into the representation of component-task-specific information.

#### Task-switching within PRP trials

In studies focusing on the representation of component-task-specific information, there are two potentially relevant tasks, Task A and Task B (i.e., letters represent placeholders for tasks), and the sequence of these two tasks is manipulated within a PRP trial. As a result, there are PRP trials in which T2 differs from T1 (e.g., A as T1 and B as T2 [i.e., AB]) and PRP trials in which the same task is performed as T1 and T2 (e.g., BB; see Table 1). The first trial type is referred to as a T1-T2 switch trial because the task switches from T1 to T2 and the second trial type as a T1-T2 repetition trial because T1 is repeated in T2. With this paradigm, performance in T2 is typically worse in T1-T2 switch trials than in T1-T2 repetition trials, an effect referred to as T1-T2 switch costs.

**Table 1.** Task sequences at the level of Task 1 (T1) and Task 2 (T2) within a PRP trial for the combination of the two-component tasks A and B.

Task sequence		T1-T2 sequence within a trial
**T1**	**T2**	
A	A	repetition
B	B	repetition
A	B	switch
B	A	switch

For instance, in a study by Lien and colleagues (2003), participants performed a letter categorisation task (i.e., vowel vs. consonant) and a digit categorisation task (i.e., odd vs. even). In T1-T2 repetition trials, either the letter categorisation task or the digit categorisation task was repeated across T1 and T2, whereas in T1-T2 switch trials, there was a switch either from the letter categorisation task in T1 to the digit categorisation task in T2 or from the digit categorisation task in T1 to the letter categorisation task in T2. In addition to a PRP effect, this study showed that T2 performance was worse in T1-T2 switch trials than in T1-T2 repetition trials, reflecting switch costs in RT2 (i.e., 50 ms in Experiment 1 and 2% in Experiment 2), which have been replicated by several subsequent studies (e.g., Band & van Nes, 2006; Hirsch, Nolden, Declerck et al., 2018).

#### Empirical findings and theoretical implications for component-task-specific information

To account for switch costs in T2, researchers made use of theories originating from the task-switching domain. In task-switching research, switch costs are a well-established finding. In contrast to dual-task studies, in task-switching studies, subjects perform only one task per trial, and the relevant task repeats or switches across two consecutive trials (e.g., Hirsch et al., 2016; Meiran, 1996; Rogers & Monsell, 1995; see e.g., Koch & Kiesel, 2022, for a review). The cognitive representation of a single task is referred to as the “task set,” and switch costs are explained by assuming that two task sets cannot simultaneously be held active in working memory, at least not at the same degree of activation. Consequently, when a task switches, the cognitive system has to be reconfigured in accordance with new task demands, a process termed task-set reconfiguration (e.g., Meiran, 1996). Task-set reconfiguration includes goal shifting (Rubinstein et al., 2001), stimulus and response set biasing (Meiran, 2000), and/or the retrieval of S-R mappings from long-term memory (Mayr & Kliegl, 2000). Moreover, researchers theorised that the activation of a task set persists even after the completion of the task. This persisting activation leads to interference when a new task becomes relevant. This is because the persisting activation (positively) primes the old task set, and prior inhibition of the currently relevant task set leads to negative priming of this task set (e.g., Allport et al., 1994; Allport & Wylie, 1999).

According to these accounts, switch costs in T2 of a PRP trial suggest that a new task set has to be implemented in working memory to perform T2 and that task-set reconfiguration, along with the resolution of proactive interference, might be required to enable T2 processing in T1-T2 switch trials. Consequently, T2 switch costs indicate that information relevant for the processing of T1 (i.e., T1 task set) is separately represented from the information required to perform T2 (i.e., T2 task set). Importantly, switch costs in T2 did not significantly differ across the SOAs (e.g., Band & van Nes, 2006; Lien et al., 2003). The additivity of the effects of task transition and SOA suggests task-set reconfiguration and/or proactive interference does not contribute to the PRP effect (see e.g., Lien et al., 2003, for a response-selection bottleneck model incorporating this notion).

#### Summary

Empirical evidence suggests that information for T1 processing is separately represented from information for T2 processing. Thus, there are two separate representations, namely the T1 task set for T1 information and the T2 task set for T2 information.

### Representations of component-task order information and their control

Studies addressing the question of how the order of the two component tasks is controlled in dual-task contexts allow for further conclusions about the nature of a dual-task representation. These studies typically used the order-switching paradigm.

#### Order switching across PRP trials

The order-switching paradigm requires two tasks (e.g., A and B) and two component-task orders (i.e., Order 1 with Task B as T1 and Task A as T2 [i.e., AB] and Order 2 with BA). In this paradigm, there is always a task switch across T1 and T2 (i.e., T1-T2 switch trial). Thus, the T1-T2 sequence is constant across PRP trials because T1 always differs from T2. In contrast, the component-task order varies across PRP trials, resulting in same-order trials and different-order trials (e.g., Kübler et al., 2018, 2022a; Luria & Meiran, 2003, 2006; Strobach et al., 2019, 2021). In different-order trials, the order of the two component tasks in a given trial *n* differs from the component task order in the previous trial *n* − 1 (e.g., trial *n* − 1 with AB & trial *n* with BA [i.e., AB → BA] or BA → AB). In same-order trials, the component task order in a given trial *n* is identical to that in the previous trial *n* − 1 (e.g., BA → BA or AB → AB; see Table 2). Typically, performance in both T1 and T2, as indicated by RT and error rates, is worse in different-order trials than in same-order trials, reflecting order switch costs (e.g., Kübler et al., 2018; Luria & Meiran, 2003, 2006; see also Strobach et al., 2024a, for a review on sequential effects in task-order switch costs).

**Table 2.** Task sequences in the order-switching paradigm at the level of Task 1 (T1) and Task (T2) in a current trial *n* and the previous trial *n* − 1 and the resulting T1-T2 sequence within a trial, component-task order sequence across trials, T2-T1 sequence across trials, and T1-T1 sequence across trials.

Task sequences				T1-T2 sequence with a trial	Order sequence across trials	T2-T1 sequence across trials	T1-T1 sequence across trials
**Trial** *n* − **1**		**Trial *n***					
**T1**	**T2**	**T1**	**T2**				
A	B	A	B	switch	repetition	switch	repetition
B	A	B	A	switch	repetition	switch	repetition
A	B	B	A	switch	switch	repetition	Switch
B	A	A	B	switch	switch	repetition	Switch

For instance, in a seminal study by Luria and Meiran (2003), participants performed a letter categorisation task (i.e., B vs. D) and a colour categorisation task (i.e., blue vs. pink rectangle). There were two possible component-task orders. In Order 1, T1 was the letter task and T2 the colour task, whereas in Order 2, T1 was the colour task and T2 the letter task. A cue at the beginning of each trial indicated the relevant component-task order. A white square introduced the *colour then letter order* and an arrow *the letter then colour order* or vice versa. In different-order trials, participants switched from the *colour then letter order* in trial *n* − 1 to the *letter then colour order* in trial *n* or from the *letter then colour order* in trial *n* − 1 to the *colour to letter order* in trial *n*. In same-order trials, they executed either the *colour then letter order* or the *letter then colour order* in both trial *n* − 1 and trial *n*. Across three experiments, Luria and Meiran (2003) observed worse performance in different-order trials than in same-order trials, reflecting order switch costs (i.e., 124 ms and 2.2% in T1 and 117 ms in T2 in Experiment 2).

Note that in order-switching studies, there is a confound for T1 between the component-task order sequence and the sequence between T2 in the previous trial *n* − 1 and T1 in a given trial *n*. That is, different-order trials are accompanied by a T2-T1 repetition across PRP trials, meaning that participants perform the same task as T1 in a given trial *n* and T2 in the previous trial *n* − 1 (e.g., AB → *B*A). In contrast, a same-order trial is linked to a T2-T1 switch across PRP trials (e.g., BA → *B*A; see Table 2). Thus, T1 in a given trial *n* always differs from T2 in the previous trial *n* − 1.

Studies in which participants repeat and switch single tasks at a trial-by-trial level (i.e., task-switching studies) showed that switching between tasks impairs performance relative to repeating a task, as evident in increased RTs and error rates (e.g., Hirsch. Nolden, Declerck et al., 2018; Koch et al., 2023 see e.g., Koch et al., 2018; Koch & Kiesel, 2022; Vandierendonck et al., 2010 for reviews). Thus, the task switch between T2 in trial *n* − 1 and T1 in trial *n* should result in worse T1 performance in same-order trials, which comprise a T2-T1 switch, than in different-order trials, which comprise a T2-T1 repetition. The finding of order switch costs suggests that the effect of switching the order of the component tasks across PRP trials is stronger than that of switching component tasks across T2 and T1.

#### Empirical findings and theoretical implications about component-task order information

Order switch costs suggest that the processing order of the two component tasks is controlled by order representations, called “order sets.” It has been theorised that the order set is an explicit representation containing information on the processing order of the component tasks only, analogously to a do-to list (e.g., first A, then B; Kübler et al., 2022b; see, however, also Huestegge et al., 2021), and that it is responsible for activating the component tasks according to the planned order. Thus, order sets do not include information specific to the component tasks, such as information on the relevant stimuli, responses, and S-R mappings (i.e., T1 and T2 task sets).

The finding of order switch costs indicates that order control is not solely based on “a first-come first-served” basis, as proposed by the structural response-selection bottleneck model (Pashler, 1994), but that additional cognitive task representations (i.e., order sets) guide the access to the capacity-limited stage of response selection (e.g., Kübler et al., 2018). Several studies provide evidence that order-set control relies on top-down cognitive processes.

For example, in a study by Kübler and colleagues (2022a, Experiment 1), participants performed a visual letter categorisation task and an auditory tone-categorisation task in each trial. In addition to the component-task order sequence, the authors varied the working memory load between blocks. In a high-load condition, there were four stimuli and responses for each component task, resulting in eight S-R mappings. In a low-load condition, only two stimuli for each component task were presented. Thus, participants had to maintain only four S-R mappings in working memory. Kübler and colleagues (2022a) observed that order switch costs in T1 and T2 were reduced when working memory load was increased. The authors concluded that increased working memory load hampered the active maintenance of order sets in working memory and, therefore, the repetition priming effect in same-order trials was less pronounced, thus reducing order switch costs.

Other studies observed that order switch costs were reduced when subjects had the opportunity to prepare for the upcoming task in advance, further confirming the involvement of top-down cognitive control processes in task-order control. For instance, in a study by Luria and Meiran (2003), the component-task order sequence was random, but order cues were presented before the onset of the task stimuli to indicate the relevant component-task order. A manipulation of the time interval between the cue and the stimulus for T1 (cue-target interval) showed that order switch costs in T1 and T2 were reduced with long preparation time compared to short preparation time. The preparatory reduction of order switch costs indicates that participants used the preparation time to activate the new order set in working memory (see task-set reconfiguration models in task-switching research; e.g., Meiran, 1996; Rogers & Monsell, 1995).

In contrast to the study by Luria and Meiran (2003), in which the component-task order was random, and participants had to rely on order cues to prepare for the component-task order, Strobach and colleagues (2019) used a different approach to investigate the effects of preparation on order switch costs. In their study, there were random order blocks without order cues and systematic-order blocks with an instructed order of the component tasks. As a result, the component-task order was predictable. Besides the predictability of the upcoming order, Strobach and colleagues (2019) varied the inter-trial interval (ITI) between two PRP trials. The ITI reflects the time interval between the response for T2 in a given trial and the onset of the stimulus for T1 in the next trial. The authors found that order switch costs in T1 and T2 were reduced in predictable order blocks relative to random order blocks. In addition, the reduction of order switch costs in predictable order blocks compared to random blocks occurred only with long ITIs but not with short ITIs. These findings suggest that participants prepared for the upcoming order when the order sequence was predictable and that preparation based on order predictability takes time. Besides these behavioural studies, brain imaging studies showed a stronger activation of prefrontal cortical regions, a neuroanatomical correlate of top-down control processes, in different-order trials than in same-order trials (e.g., Szameitat et al., 2006; see also Kübler et al., 2019; Strobach, Soutschek et al., 2015).

#### Summary

Order-switching studies suggest that the representation of a dual task is a complex representational structure of different components, including a task-order set, a T1 task set, and a T2 task set (Kübler et al., 2022a, 2022b). These representational components are assumed to be separately adjustable to dual-task demands (Kübler et al., 2022a).

### Representations of dual-task identity information and their control

To examine the cognitive representation of a dual task, Hirsch and colleagues (2017) developed a different empirical approach, which they called the task-pair switching logic. The authors incorporated this logic into the PRP paradigm.

#### Task-pair switching across PRP trials

In contrast to the previous two paradigms (i.e., T1-T2 switching within PRP trials and component-task order switching across PRP trials), the task-pair switching logic requires three tasks (e.g., A, B, and C), which are combined into two task-pairs. In each given PRP trial, only one task pair has to be performed. T1 is constant across task-pairs, whereas T2 varies across task-pairs or vice versa (e.g., CA and CB; see Table 3).^3^ Note that each task-pair comprises a task switch across T1 and T2 within a PRP trial (e.g., CA and CB). Thus, at the level of the component tasks, there is always a T1-T2 switch within a PRP trial. However, the sequence of the task-pairs varies across PRP trials, resulting in task-pair switch trials and task-pair repetition trials. In task-pair switch trials, the task-pair in a given PRP trial differs from the task-pair in the previous PRP trial (e.g., CA → CB or CB → CA), whereas in task-pair repetition trials, the task-pair is identical across two consecutive PRP trials (e.g., CB → CB or CA → CA).

**Table 3.** Task sequences in the task-pair switching logic at the level of Task 1 (T1) and Task (T2) in a current trial *n* and the preceding trial *n* − 1 and the resulting task-pair sequence across trials, T1-T2 sequence within a trial, T2-T1 sequence across trials, and T1-T1 sequence across trials.

Task sequences				Task-pair sequence across trials	T1-T2 sequence within a trial	T2-T1 sequence across trials	T1-T1 sequence across trials
**Trial *n* − 1**		**Trial *n***					
**T1**	**T2**	**T1**	**T2**				
**Constant T1 and varying T2**							
C	A	C	A	repetition	switch	switch	repetition
C	B	C	B	repetition	switch	switch	repetition
C	A	C	B	switch	switch	switch	repetition
C	B	C	A	switch	switch	switch	repetition
**Varying T1 and constant T2**							
A	C	A	C	repetition	switch	switch	repetition
B	C	B	C	repetition	switch	switch	repetition
A	C	B	C	switch	switch	switch	switch
B	C	A	C	switch	switch	switch	switch

**Figure 2.**
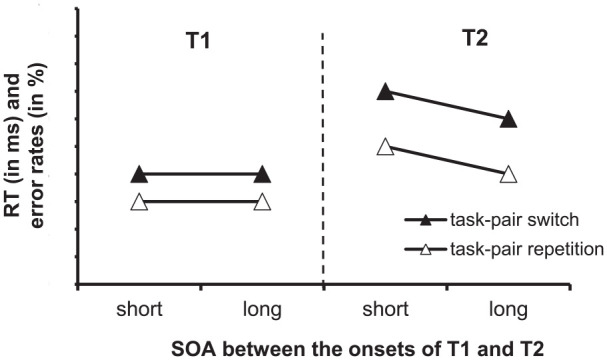
Idealised data for a task-pair switching experiment with reaction times (RT) in ms and error rates in % for Task 1 (T1) and Task 2 (T2) as a function of the task-pair sequence (task-pair switch vs. task-pair repetition) and the SOA (stimulus-onset-asynchrony) between the onsets of the stimulus for T1 and the stimulus for T2; short vs. long).

With the task-pair switching logic, performance in T1 and T2, as evident in RTs and error rates, is typically worse in task-pair switch trials than in task-pair repetition trials, resulting in task-pair switch costs. Figure 2 illustrates idealised data. Task-pair switch costs are a stable finding, which is observable with various numbers of task-pairs (e.g., 2 vs. 3; Hirsch et al., 2017; Hirsch, Nolden, Philipp, & Koch, 2018), different task types (e.g., T1 requiring speeded manual responses or deferred non-speeded vocal responses; Hirsch et al., 2017; Koch & Rumiati, 2006, Experiment 3), and different degrees of overlap in T1 and T2 responses (e.g., conceptual vs. physical overlap; e.g., Hirsch et al., 2017).

For instance, in a study by Hirsch, Nolden, Philipp and colleagues (2018, Experiment 2), the stimulus material consisted of a high-pitch tone, a low-pitch tone, and pictures showing a can or a cup with a handle on the left or right side. The tones were linked to a pitch categorisation task (low vs. high) and the pictures to an object categorisation task (i.e., can vs. cup) and a side categorisation task (i.e., left vs. right handle). There were two task-pairs, including Task Pair 1 with the pitch task as T1 and the object task as T2 and Task Pair 2 with the pitch task as T1 and the side task as T2. Cues were the German words for object and side (i.e., Objekt & Seite). In the case of a task-pair switch, participants switched from Task-pair 1 in trial *n* − 1 to Task-pair 2 in trial *n* (i.e., pitch task as T1 and object task as T2 in trial *n* − 1 to the pitch task as T1 and the side task as T2 in trial *n*), or they switched from Task-Pair 2 in trial *n* − 1 to Task-Pair 1 in trail *n* (i.e., pitch task as T1 and side task as T2 in trial *n* − 1 to the tone task as T1 and the object task as T2 in trial *n*). In the case of a task-pair repetition, they performed either Task-pair 1 (i.e., tone task as T1 and object task as T2) or Task-pair 2 (i.e., the tone task as T1 and side task in T2) in both trial *n* − 1 and trial *n*. Hirsch, Nolden, Philipp and colleagues (2018) observed that performance was worse in task-pair switch trials than in task-pair repetition trials, resulting in task-pair switch costs of 119 ms in T1 and 184 ms and 3.3% in T2.

#### Empirical findings and theoretical implications about dual-task identity information

In task-pair switching studies, the relevant task-pair is announced by a cue at the beginning of each trial. In task-switching research, it has been found that repeating the cue itself can already lead to some perceptual repetition priming benefit relative to cue switches, suggesting that task switch costs include a perceptual priming component (e.g., Altmann, 2006). With one cue per task-pair, a task-pair switch is always accompanied by a cue switch and a task-pair repetition by a cue repetition. Accordingly, task-pair switch costs might be the result of repetition priming at the level of cue processing instead of switching task-pairs.

To exclude this alternative explanation for task-pair switch costs, Hirsch and colleagues (2021, Experiment 1) employed two cues per task-pair. Due to the use of two cues per task-pair, this study was able to measure task-pair switch costs by comparing performance in task-pair repetition trials which were accompanied by a cue switch and task-pair switch trials which are by definition linked to a cue switch. Using this 2:2 cue-to-task-pair mapping, Hirsch and colleagues (2021, Experiment 1) confirmed substantial task-pair switch costs when controlling for cue switches. The study also showed cue switch costs by contrasting task-pair repetition performance across trials with a cue switch and trials with a cue repetition. Cue switch costs might reflect priming of cue-encoding processes (see also Schneider & Logan, 2005). However, the observation of “pure” task-pair switch costs suggests that switching task-pairs per se produces a substantial cost that cannot be accounted for by cue-encoding benefits due to lower-level priming effects (see e.g., Jost et al., 2015, for a review).

Moreover, task-pair switch costs in T1 do not reflect task-set reconfiguration at the local level of the component tasks (i.e., T2 in the previous trial and T1 in a given trial). This alternative explanation can be excluded because in both task-pair switch and task-pair repetition trials, T1 in the current task-pair is accompanied by a task switch from the preceding T2 in task-pair *n* − 1 (e.g., CB → CA vs. CA → CA; see Table 3).

Ruling out these alternative explanations for task-pair switch costs supported the hypothesis that task-pair switch costs are attributable to switching processes at the level of task-pairs (e.g., Hirsch et al., 2017, 2021; Hirsch, Nolden, Philipp, & Koch, 2018) That is, task-pair switch costs suggest that information about the task-pair was activated in the previous trial and that this information persisted to a certain degree until the next trial. The persisting activation facilitated performance when the same task-pair was relevant again (i.e., transfer benefit) and hampered performance when a new task-pair was performed (i.e., transfer cost).

Following this rationale, task-pair switch costs suggest that the identity of T1 and T2 is jointly represented in a single cognitive representation, called the “task-pair set” (Hirsch et al., 2017). The task-pair set is assumed to be an explicit (separate) representation, containing only information about the identity of the dual task which in turn determines the identities of the two component tasks (Hirsch, Nolden, Philipp, & Koch, 2018). For example starting a car has the temporally overlapping component tasks of releasing the clutch and pushing down the gas pedal. In this example, the task-pair set comprises the information that the relevant dual task is to start a car by releasing the clutch and pushing down the gas pedal, but it does not include information on the cognitive and motor demands for executing these two component tasks themselves (i.e., T1 and T2 task sets).

Based on their set of dual-task studies, Hirsch et al. (2021) conceptualised the cognitive representation of a dual task as a hierarchical multi-component representation. This representation comprises a task-pair set, a T1 task set, and a T2 task set. They assumed that the task-pair set is organised at a hierarchically higher level than the component-task-specific task sets of T1 and T2. The hierarchy is defined by a temporal precedence, meaning that the identity of the task-pair has to be known to execute the appropriate component tasks (Hirsch, Nolden, Philipp, & Koch, 2018). Thus, the task-pair set has to be activated in working memory before or during the selection of the component tasks.

##### Temporal dynamics of task-pair set activation

To get insights into the point in time at which a task-pair set is implemented in working memory, Hirsch and colleagues (2021, Experiment 2) used a go/nogo-manipulation in their task-pair switching study. In this study, subjects performed both go-trials, in which the stimuli for T1 and T2 were presented after a cue, and no-go trials (cue-only trials), in which a cue announced the relevant task-pair, but the stimuli for T1 and T2 did not appear. Task-pair switch costs were comparable in size after go-trials and no-go trials. Importantly, when defining the task-pair sequence based on the task-pair following a nogo-trial and the task-pair preceding the nogo-trial instead of defining it based on the sequence across the nogo-trial and the following go-trial, task-pair switch costs were not significant. Thus, persisting activation of the task-pair performed before the nogo-trial cannot explain the task-pair switch costs after nogo-trials.

The finding of switch costs after nogo-trials speaks against the notion that task-pair sets represent solely an episodic after-effect of having performed a dual task. The notion of episodic after-effects would be in line with an (non-hierarchical) episodic-binding account (see, e.g., Frings et al., 2020; Hommel, 2004, for a similiar task-switching account), which would contradict the notion that task-pair sets are organised at a hierarchically higher level than the specific T1 and T2 task sets. This is because according to this account, task-pair switch costs would arise due to an episodic representation of the specific task-pair performed in the previous trial and would be a pure by-product of performing a dual task. Thus, task-pair switch costs would be only attributable to memory-based episodic after-effects (i.e., inertia of previously formed task-pair sets) and would not occur after nogo-trials. The finding of task-pair switch costs after no-go trials suggests that task-pair sets are intentionally implemented and maintained in working memory before the presentation of the T1 and T2 stimuli and that task-pair switch costs are a marker of proactive dual-task control.

Specifically, the task-pair cue seems to activate a higher-level task representation about the identity of the relevant dual task that enables subjects to specify the appropriate component tasks. Thus, the cue is used to retrieve (at least partly) the relevant task-pair set in working memory and the translation take less time when the corresponding task-pair set was still activated to some degree due to its relevance in the preceding trial.^4^

Additional analyses reported in the study by Hirsch, Nolden, Philipp and colleagues (2018) further confirm this conclusion. In this study, task-pair switch costs did not differ depending on whether the SOA from the previous trial was repeated or changed relative to the current trial (i.e., SOA sequence). Thus, the authors found no evidence for the notion that information about the previous SOA duration is part of the task-pair set, suggesting that task-pair sets do not include temporal information about the onset of T2. If task-pair sets were a pure by-product of performing a dual task and were available only after executing a dual task, task-pair sets would probably include information about the SOA duration in the previous trial. In this case, task-pair switch costs should be affected by the SOA sequence. The finding that the SOA sequence did not modulate task-pair switch costs supports the notion that task-pair sets are activated by the cue at the beginning of a dual-task trial. As the SOA duration is unknown at this point in time, information about the SOA duration is not included in task-pair sets.

Note, however, that the existence of task-pair switch costs after nogo-trials and the absence of a modulation of task-pair switch costs by the SOA sequence do not exclude the possibility that, in addition to task-pair set activation before dual-task execution, an episodic representation of the previous task-pair is created after the execution of a dual task. Moreover, there was no systematic variation of the SOA duration in the existing task-pair switching studies. As a consequence, more research is needed to elucidate the point in time at which task-pair set activation is initiated.

##### Task-pair set selection

An important question is whether the control of task-pair sets involves inhibiting task-pairs in addition to activating them. To examine this question, Hirsch et al. (2017) combined the task-pair switching logic with the “backward-inhibition” paradigm (see e.g., Mayr & Keele, 2000, for backward-inhibition in task-switching research and Koch et al., 2010, for a review on this topic). In this study, subjects performed three task-pairs, and performance was contrasted across *n* − 2 task-pair repetition trials and *n* − 2 task-pair switch trials. In *n* − 2 task-pair switch trials, the task-pair in trial *n* differed from the task-pair in trial *n* − 2 (e.g., *task-pair 3* → task-pair 2 → *task-pair 1*), whereas in *n* − 2 task-pair repetition trials, the task-pair was identical across trial *n* − 2 and trial *n* (e.g., *task-pair 1* → task-pair 2 → *task-pair 1*). Importantly, in both *n* − 2 task-pair switch trials and *n* − 2 task-pair repetition trials, there was a task-pair switch between trial *n* − 2 and trial *n* − 1 as well as between trial *n* − 1 and trial *n*. Performance differences across *n* − 2 task-pair switch trials and *n* − 2 task-pair repetition trials can appear in the form of *n* − 2 task-pair repetition costs or *n* − 2 task-pair repetition benefits.

The *n* − 2 repetition costs (i.e., worse performance in *n* − 2 task-pair repetition trials than in *n* − 2 task-pair switch trials) would reflect persisting after-effects of task-pair inhibition (see e.g., Koch et al., 2010, for a review). More precisely, these costs would indicate that in *n* − 2 repetition trials, subjects switch to a task-pair which was inhibited when switching from the task-pair in trial *n* − 2 to that in trial *n* − 1. In contrast, in *n* − 2 switch trials, subjects switch to a task-pair which was not relevant in trial *n* − 2 and, therefore, no inhibition of this task-pair was needed in trial *n* − 1.

In contrast, *n* − 2 repetition benefits, with worse performance in *n* − 2 switch trials than in *n* − 2 task-pair repetition trials, would provide evidence for persisting task-pair set activation, which results in residual positive priming of task-pairs. The rationale is that the residual activation, and hence, also the priming effect is stronger the more recently the task-pair has been performed. In *n* − 2 task-pair repetition trials, the target task-pair has been performed two trials ago, whereas in *n* − 2 task-pair switch trials, it has been performed more than two trials ago. As a result, the beneficial impact of persisting task-pair set activation should be more pronounced in *n* − 2 task-pair repetition trials than in *n* − 2 task-pair switch trials.

Hirsch and colleagues (2017) observed an *n* − 2 repetition benefit across three experiments with different degrees of overlap in the responses of T1 and T2 (i.e., conceptual vs. physical response-set overlap). This benefit suggests that switching to a new task-pair is not accompanied by inhibiting the last task-pair set, so that its residual activation persists even after the completion of a dual task. When the task-pair is repeated, the persisting activation facilitates performance because it positively primes the relevant task-pair (see also Allport et al., 1994; Allport & Wylie, 1999).^5^

In line with the go/nogo task-pair switching study which revealed task-pair switch costs after nogo-trials, it has been suggested that in addition to this task-pair set “inertia,” task-pair switch costs reflect processes involved in reconfiguring the cognitive system in accordance with new dual-task demands (e.g., Hirsch et al., 2017, 2021; Hirsch, Nolden, Philipp, & Koch, 2018). When a task-pair repeats, the previous task-pair set can be employed again. In the case of a task-pair switch, a task-pair set reconfiguration is required to implement the new task-pair set in working memory. Reconfiguration can be understood as a gradual process, representing a bias that favours the processing of one task-pair rather than the processing of the competing task-pair (see Meiran, 2000, for a similar notion in task switching).

The cognitive processes involved in task-pair set reconfiguration and/or the resolution of proactive interference seem to act independently of the processes reflected by SOA effects. This is because the task-pair sequence usually does not affect SOA effects in T1 or T2 (e.g., Hirsch et al., 2017, 2021; Hirsch, Nolden, Philipp, & Koch, 2018). This suggests that task-pair set reconfiguration occurs before response selection for T1.

**Figure 3.**
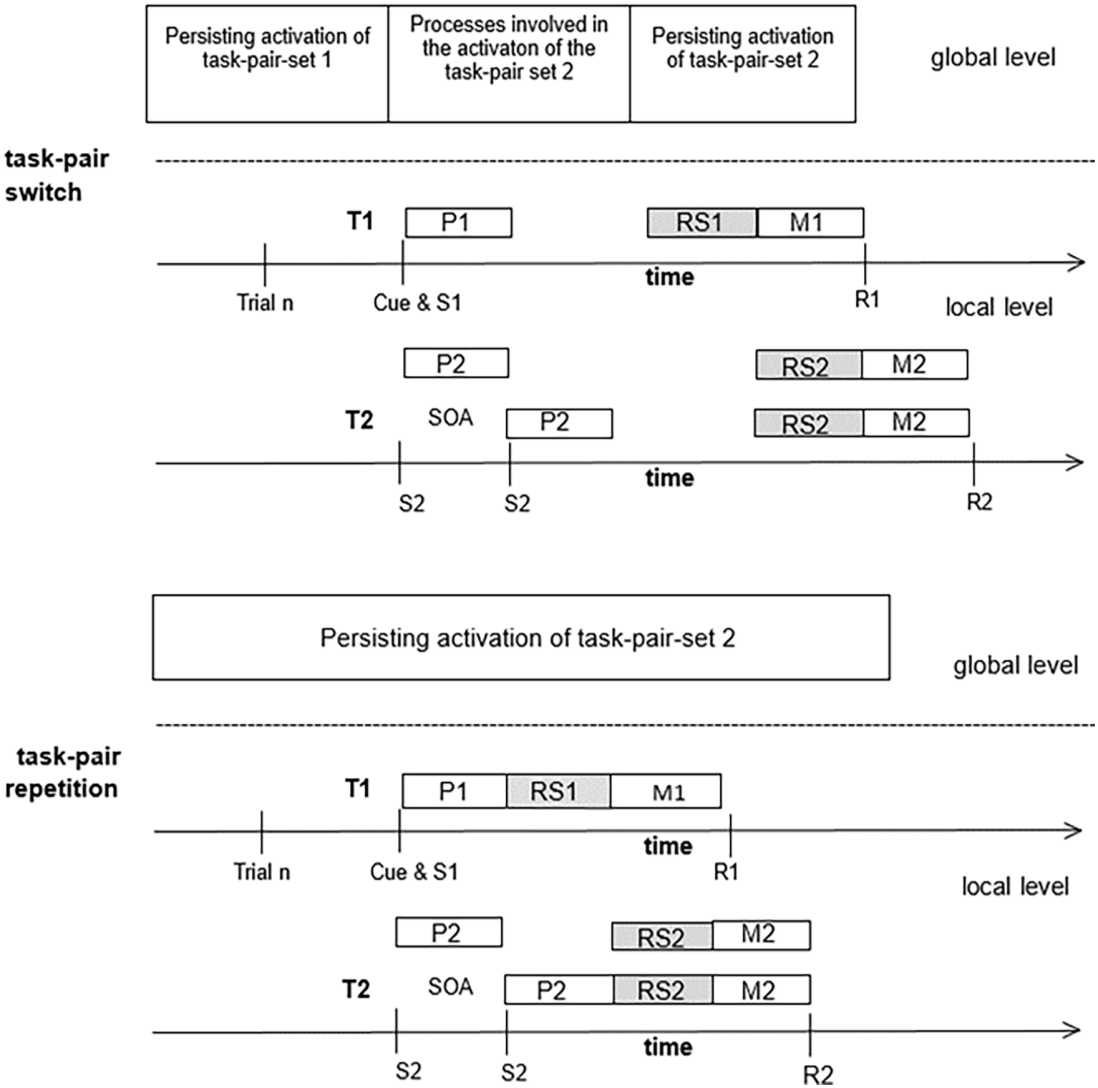
Task-pair set control and its effects on T1 performance. *Note.* SOA = stimulus onset asynchrony, P1 and P2 = perceptual processes for Task 1 (T1) and Task 2 (T2), RS1 and RS2 = response selection for T1 and T2, M1 and M2 = motor processes for T1 and T2; S1 and S2 = stimulus for T1 and T2, R1 and R2 = response for T1 and T2.

Taking this finding into account, task-pair switch costs in T1 can be explained by assuming that in task-pair switch trials, the selection of the T1 task set, which is a prerequisite for T1 response selection, is prolonged for the time needed to reconfigure the cognitive system with the new task-pair demands (see Figure 3).^6^ Alternatively, task-pair set reconfiguration can proceed in parallel with the selection of the T1 task set, leading to a prolongation of these processes. According to these ideas, task-pair switch costs in T1 reflect the duration of active cognitive control processes. Passive (automatic) memory-based after-effects of previous active control processes might additionally contribute to task-pair switch costs (Hirsch et al., 2017).

#### Summary

Task-pair switching studies suggest a number of theoretical implications: The execution of a dual task presupposes the activation of a cognitive dual-task representation in working memory. The cognitive representation of a dual task can be conceptualised as a hierarchical multi-component representation with different components. Within this representation, task-pair sets are organised at a hierarchically higher level than the component-task-specific task sets of T1 and T2. This is because the task-pair set comprises information on the T1 and T2 identities and, therefore, has to be available before activating the corresponding T1 and T2 task sets. When a task-pair changes, the cognitive system has to be reconfigured in accordance with the new dual-task demands, and the selection of task-pair sets is supported by persisting activation which results in residual positive priming of previous task-pairs.

### Integrating the findings about the different components of a dual-task representation

So far, the representation of component-task-specific information, component-task order information, and dual-task identity information have been predominantly examined in independent research lines (e.g., Hirsch et al., 2021; Luria & Meiran, 2006; see e.g., Kübler et al., 2022b, for an exception). To integrate the findings observed in these research lines, it is essential to consider all these components when conceptualising the cognitive representation of a dual task. Consequently, the cognitive representation of a dual task should incorporate a task-pair set, an order set, a T1 task set, and a T2 task set.

**Figure 4.**
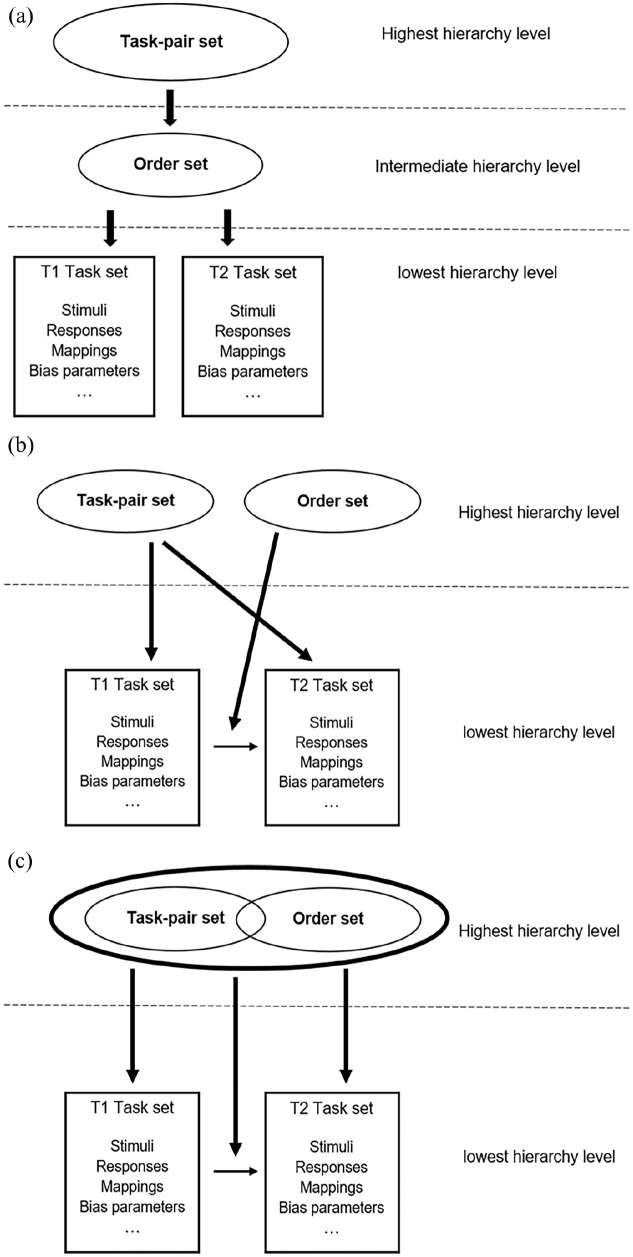
Three possible conceptualisation of a multi-component hierarchical dual-task representation, including a task-pair set, an order-set, and the Task 1 (T1) and Task 2 (T2) task sets: (a) Dual-task representation with three hierarchy levels and independent task-pair sets and order sets; (b) Dual-task representation with two hierarchy levels and independent task-pair sets and order sets; (c) Dual-task representation with two hierarchy levels with dependent task-pair sets and order sets.

One possible conceptualisation of a dual-task representation is a hierarchical multi-component representation, comprising a task-pair set, an order set, and the T1 and T2 task sets at different hierarchical levels (see Figure 4A). Specifically, it is conceivable that the task-pair set is organised at the hierarchically highest level, followed by the order set at the intermediate hierarchy level, and the two-component-task-specific task sets of T1 and T2 at the lowest hierarchy level. The hierarchy is defined as a temporal precedence of particular information over other information. To perform a dual task, we first have to specify the identity of the dual task (i.e., starting a car) with its component tasks before we can determine the order of the component tasks. Hence, the availability of information about the component-task identity is a prerequisite for the definition of the component-task order, meaning that task-pair set reconfiguration and order-set reconfiguration occur sequentially and independently of each other. Only if the relevant component tasks and their processing order are known, the appropriate T1 and T2 task sets can be selected in the relevant order (see also Kübler et al., 2022a).

Alternatively, to a dual-task representation with three hierarchical levels, it is also conceivable that dual-task representations comprise only two hierarchy levels. Specifically, the task-pair set and the order set might be organised at the same level but as two independent components and the two task sets for T1 and T2 might be organised at a subordinate hierarchy level (Figure 4B). With such an organisation of the components of a cognitive dual-task representation, there is no temporal precedence of task-pair information over component-task-order information. Rather task-pair information and order information are activated simultaneously. Thus, task-pair set and order-set reconfiguration occur in parallel but independently of each other. The task-pair set indicates the relevant component tasks, whereas the order set determines which component task has to be performed as T1 and which as T2. Note, however, that with such a conceptualisation of a cognitive dual-task representation, cognitive processes are required to integrate the information on the task-pair and the component-task order.

Finally, it is also possible that the cognitive representation of a dual task is organised at two hierarchy levels, with a “meta-task set” at the hierarchically highest level and the two task sets for T1 and T2 at the subordinate level (see Figure 4c). The meta-task set includes task-pair information and component-task order information as two dependent subcomponents. Hence, the reconfiguration of the task-pair set, the order set, or both results in a new meta-task set.

All three conceptualisations of a dual-task representation are conceivable. The current studies on the nature of dual-task representations do not allow differentiating between these three alternatives because the representation of component-task-specific information, component-task order information, and dual-task identity information has been predominantly studied in independent research lines. Thus, more research is needed to test the models against each other. In the first step, researchers could combine task-pair sequence, component-task-order sequence, and T1-T2 sequence manipulations within an experiment to examine whether these manipulations result in independent effects. For instance, a serial and independent reconfiguration of task-pair sets and order sets should result in additive effects of the task-pair sequence and the component-task order sequence (see Philipp & Koch, 2010; Vandierendonck et al., 2008 for the use of the two-componential switching paradigm in task-switching research). In addition, a computational approach could help to identify the best model.

## The role of transfer in research on dual-task representations

The majority of the studies addressing the nature of dual-task representations analysed the performance on a trial-by-trial level (or task-by-task level in studies on the representation of component-task-specific information, e.g., Band & van Nes, 2006) and examined the effects dual-task demands in the previous trial on the performance in a given trial. The different types of switch costs elucidated in the present review (i.e., T1-T2 component task switch costs, task-order switch costs, and task-pair switch costs) indicate that in each trial, an abstract representation of information and/or cognitive parameters relevant to a dual task, also called control structure, is implemented in working memory to meet the current dual-task demands. The finding that performance in a given dual task is affected by the dual-task demands in the previous trial represents a carry-over effect, which can be understood as a type of transfer. Transfer means that in the case of a component-task repetition, component-task-order repetition, and/or task-pair repetition, the implemented cognitive task representation can be applied to the next dual task, leading to transfer benefits. In the case of a change in one of these components, a new cognitive task representation has to be implemented in working memory, and, therefore, there is a transfer cost.

In sum, the reviewed studies showed that transfer effects occur at different representational levels. An interesting question is whether the transfer process itself is also abstract. The transfer process can be defined as abstract, when it is not specific for a certain combination of component tasks and/or a certain processing order of the component tasks. In other words, the transfer process is abstract, when practicing a dual task with a specific pair of component tasks also leads to performance improvements in terms of reduced T1-T2 switch costs, order switch costs, and task-pair switch costs for a dual task with other component tasks.

Dual-task practice studies can help to answer the question about the abstractness of the transfer process. However, so far, studies primarily focused on the effect of practice on dual-task costs and the PRP effect (see e.g., Schubert et al., 2024; Strobach, 2020, for reviews). These studies showed that practicing a dual task results in a stronger decrease of dual-task interference than practicing the component tasks of a dual task in single-task conditions (e.g., Kramer et al., 1995; Schubert et al., 2017). These findings indicate that the dual-task practice effect is not due to learning the decision process of a specific component task, but that practice might result in efficient allocation of limited central processing resources to the two tasks (see e.g., Strobach, 2020, for reviews). Further studies demonstrated that these skills are, at least to some extent, transferable to dual-task contexts with other component tasks, indicating that participants learn to deal flexibly with dual-task demands (e.g., Liepelt et al., 2011; Schubert et al., 2017).

In contrast to studies on practice-related reductions in dual-task costs and in the PRP effect, a recent practice study by Strobach (2024b) examined the effect of practice on order switch costs. In this study, participants attended either four single-task practice sessions or four dual-task practice sessions with a random order of the component tasks. Strobach (2024b) observed that in the dual-task practice group, order switch costs decreased from 6.5% in Session 1 to 1.1% in Session 4, indicating that task-order control improves during dual-task practice. However, it remains uncertain whether such practice effects also occur in PRP trials with unpracticed component tasks and whether there is a practice-related reduction in task-pair switch costs as well. Thus, existing dual-task studies do not allow for conclusions regarding the transfer process at the level of task-pair sets and order sets, indicating that more research is needed to shed light into the abstractness of the transfer process.

## Summary and conclusions

Performing two tasks in temporal overlap belongs to the most frequently investigated phenomena in cognitive research (e.g., Koch et al., 2018). There are numerous studies on dual-task interference at the level of response selection, whereas the nature of dual-task representations and the cognitive mechanisms supporting the selection of these representations have received comparably less research attention.

To get insights into the nature of dual-task representations and their control, numerous dual-task studies analysed the performance on a trial-by-trial level and examined the effects of dual-task demands in the previous trial on the performance in the next dual task. The types of switch costs elucidated in the present review (i.e., T1-T2 component task switch costs, task-order switch costs, and task-pair switch costs) indicate that in each trial, an abstract representation of the information and/or cognitive parameters needed to perform a dual task is implemented into working memory to meet the current dual-task demands. The finding that performance in a given dual task is affected by the dual-task demands of the previous trial represents a carry-over effect which can be understood as a type of transfer. Transfer means that the implemented representation can be applied to the next dual task, when the dual-task demands remain constant. Such a situation results in transfer benefits. In the case of a change in the dual-task demands, a new representation has to be implemented in working memory, leading to transfer costs.

The integration of the research lines on the representation of component-task-specific information, component-task order information, and dual-task identity information implies that a dual-task is represented as a hierarchical multi-component representation, in which certain components define the subsequent processing steps. The present review highlights the value of an integrative view on different informational components of a dual-task representation and illustrates how transfer effects can be used to study higher-order abstract representations of tasks.

## Notes

1. The perceptual and response-execution stages can proceed in parallel, if the tasks do not produce perceptual masking effects in stimulus encoding and if they do not use the same effectors.

2. We use the term “cognitive task representation” to refer to task representations in general. The term task-set is used for the cognitive representation of a single task. For dual tasks, we distinguish between T1 and T2 task sets (i.e., information about the task goals and the S-R mappings for these tasks), order sets (i.e., information about the order in which the two-component tasks have to be performed), and task-pair sets (i.e., information about the identity of the two component tasks of a dual task). Order sets and task-pair sets will be discussed in more detail later in this review.

3. Task-pair switch costs are observable with both variants of the task-pair switching logic. However, when T1 is constant across task-pairs, an additional analysis is required to show that task-pair switch costs also arise when controlling for persisting activation of T1 across task-pairs. In detail, with this variant, performance in task-pair switch trials might be worse because in task-pair switch trials, T1 in the current trial differs from the T1 in the previous PRP trial (e.g., AC in trial *n* − 1 and BC in trial *n*), whereas in task-pair repetition trials, there is a T1 repetition across two consecutive PRP trials (e.g., AC in trial *n* − 1 and AC in trial *n*). Thus, the activation of the T1 task set might persist to the next trial, even though there is the intervening T2. However, additional analyses suggest that task-pair switch costs observed with this variant are not due to such a T1 priming effect because this performance costs even arise if there is high interference between T1 and T2 due to physically overlapping response keys (i.e., same response keys for T1 and T2). Such a high interference between T1 and T2 is assumed to make an inhibition of the T1 task set necessary (see Hirsch et al., 2017).

4. Note that due to the use of one cue per task-pair the study could not exclude carry-over effects due to an automatic cue retrieval. Thus, carry-over effects might, in addition to the intentional activation of task-pair sets in working memory, contribute to task-pair switch costs after nogo-trials.

5. It is an open question why inhibition plays a different role in the selection of task-pair sets and the selection of individual task sets in single-task contexts. It is conceivable that the inhibition of a task-pair set has an impact on the activation levels of the corresponding T1 and T2 task sets. For instance, the inhibition of the task-pair set “AB” (i.e., the information that task A and B were relevant in the preceding trial) might spill over from the task-pair set to the individual task sets of task A and task B. The inhibition of a task-pair set would then have an inhibitory effect on the T1 and T2 sets, too. As either T1 or T2 remains constant across task-pairs, there could be an additional cost due to the need to overcome the inhibition.

6. In Figure 3, for the sake of simplicity, we conceptualised task-pair set reconfiguration as a separate processing stage. Note that we do not assume that there is a strict seriality of task-pair set reconfiguration and T1 task-set selection on the one hand and of perceptual processes, response selection, and response execution on the other hand. We, however, argue that there are cognitive processes that start earlier than other processes (see also Levy & Pashler, 1995).
